# Prediction of all-cause mortality in coronary artery disease patients with atrial fibrillation based on machine learning models

**DOI:** 10.1186/s12872-021-02314-w

**Published:** 2021-10-16

**Authors:** Xinyun Liu, Jicheng Jiang, Lili Wei, Wenlu Xing, Hailong Shang, Guangan Liu, Feng Liu

**Affiliations:** 1 Soochow University, Suzhou, 215006 Jiangsu People’s Republic of China; 2 Department of Cardiology, Zhengzhou University People’s Hospital, Zhengzhou, 450003 Henan People’s Republic of China; 3 Henan Key Laboratory of Chronic Disease Management, Zhengzhou, 451450 Henan People’s Republic of China; 4 Big Data Center for Cardiovascular Disease, Fuwai Central China Cardiovascular Hospital, Zhengzhou, 451450 Henan People’s Republic of China; 5 Department of Medical Imaging, Suzhou Kowloon Hospital, Shanghai Jiaotong University School of Medicine, Suzhou, 215028 Jiangsu People’s Republic of China; 6 Department of Cardiology, Suzhou Kowloon Hospital, Shanghai Jiaotong University School of Medicine, No. 118 Suzhou Industrial Park Wansheng Street, Suzhou, 215028 Jiangsu People’s Republic of China

**Keywords:** Machine learning, All-cause mortality, Coronary artery disease, Atrial fibrillation

## Abstract

**Background:**

Machine learning (ML) can include more diverse and more complex variables to construct models. This study aimed to develop models based on ML methods to predict the all-cause mortality in coronary artery disease (CAD) patients with atrial fibrillation (AF).

**Methods:**

A total of 2037 CAD patients with AF were included in this study. Three ML methods were used, including the regularization logistic regression, random forest, and support vector machines. The fivefold cross-validation was used to evaluate model performance. The performance was quantified by calculating the area under the curve (AUC) with 95% confidence intervals (CI), sensitivity, specificity, and accuracy.

**Results:**

After univariate analysis, 24 variables with statistical differences were included into the models. The AUC of regularization logistic regression model, random forest model, and support vector machines model was 0.732 (95% CI 0.649–0.816), 0.728 (95% CI 0.642–0.813), and 0.712 (95% CI 0.630–0.794), respectively. The regularization logistic regression model presented the highest AUC value (0.732 vs 0.728 vs 0.712), specificity (0.699 vs 0.663 vs 0.668), and accuracy (0.936 vs 0.935 vs 0.935) among the three models. However, no statistical differences were observed in the receiver operating characteristic (ROC) curve of the three models (all *P* > 0.05).

**Conclusion:**

Combining the performance of all aspects of the models, the regularization logistic regression model was recommended to be used in clinical practice.

**Supplementary Information:**

The online version contains supplementary material available at 10.1186/s12872-021-02314-w.

## Background

Coronary artery disease (CAD) is one of the most common types of cardiovascular diseases [1]. World Health Organization (WHO) declares that approximately 17.9 million people are died of cardiovascular diseases in 2016, accounting for 31% of all mortality [2]. CAD patients are usually complicated with atrial fibrillation (AF), which may be associated with the overlap of common risk factors between CAD and AF [3–5]. Furthermore, the occurrence of AF is closely related to the unfavorable outcomes of CAD patients, including heart failure, cerebrovascular events, acute kidney injury, and in-hospital mortality [4, 6, 7]. Even in carefully treated patients, their prognosis can be worsened by the occurrence of AF [8]. Therefore, a tool predicting all-cause mortality in CAD patients with AF is necessary for the intervention and treatment.

Machine learning (ML) is usually used to develop a predictive model to predict various results, and the computer algorithms were applied into ML to identify patterns in large databases with multiple variables [9–12]. Motwani et al. developed a ML model for the prediction of 5-year all-cause mortality in patients with only CAD [9]. Al’Aref et al. used the random forest method to accurately predict the occurrence of in-hospital death after the percutaneous coronary intervention [13]. However, a prediction model predicting all-cause mortality in CAD patients with AF has not been developed. Furthermore, the performance of different ML method models in predicting the all-cause mortality in CAD patients with AF is unclear. Herein, we aimed to develop prediction models of all-cause mortality in CAD patients with AF based on different ML methods. In addition, the performance of different ML method models was compared to obtain the optimal model.

## Methods

### Study design and population

This study was a retrospective cohort study. Patients diagnosed with CAD and AF were collected from Zhengzhou University People’s Hospital between May 2012 and July 2016. The all-cause mortality was set as the outcome indicator. There were 2050 patients recorded, and 2042 patients were complicated with CAD and AF. Among which, 5 patients were excluded due to the lack of information on outcome indicators. Finally, 2037 patients remained in this study. According to the outcome variable, patients were divided into the death group and survival group. This study protocol was approved by the Institutional Review Board of Zhengzhou University People’s Hospital and was performed in accordance with the guidelines and regulations of the Helsinki Declaration. In addition, the informed consent was provided by all participants.

### Data collection

A total of 58 patient-related variables were recorded including gender, age, number of hospitalizations, type of AF, type of CAD, diabetes mellitus, hypertension, heart failure, cardiac function, peripheral vascular disease, ischemic stroke, bleeding history, peptic-ulcer disease, drinking history, smoking history, cardioversion, percutaneous coronary intervention (PCI), CHA2DS2VASc score, HAS-BLED score, in-hospital medication (such as aspirin, warfarin, beta-blockers, etc.), in-hospital bleeding, embolization, out-of-hospital medication.

### Machine learning models

#### Variable selection

Univariate analysis was used to select predictor variables. Variables with statistical differences between the death group and the survival group were included in the predictive model.

#### Model evaluation

Three ML methods (regularization logistic regression, random forest, and support vector machines) were used to develop predictive model. The model performance was quantified by calculating the area under the curve (AUC) with 95% confidence intervals (CI), sensitivity, specificity, and accuracy.

#### Model tuning

The ML process was performed using fivefold cross-validation, a common technique in data mining currently [14]. The selection of the optimal model was based on AUC value, and the parameter corresponding to the maximum AUC value was the optimal model parameter. The optimal model parameters were as follows: (1) the regularization logistic regression model, regularization (‘l1’, ‘l2’), regularization strength (0.1, 0.3, 0.5, 1.0, 3.0, 5.0, 10.0); (2) the random forest model, the number of decision trees (10, 20, 50, 100, 200, 500), the depth of decision tree (3, 4, 5, 6); (3) the support vector machines model, kernel function (‘linear’, ‘rbf’), penalty parameter (0.01, 0.05, 0.1, 0.5, 1, 10, 50).

### Sample size and reproducibility analysis

#### Sample size

The purpose of this study was to develop models to predict all-cause mortality in CAD patients with AF. The sample size of this study was not large, therefore, whether the sample size of this study was sufficient to be evaluated by calculating the power of the model performance indicators (AUC, sensitivity, specificity, and accuracy). The power of the AUC, sensitivity, specificity, and accuracy were all 1.000, indicating that the sample size was sufficient (Additional file 1: Fig. S1).

#### Reproducibility analysis

To evaluate the reproducibility of the study, five different random number seeds were used to obtain five different training sets and test sets (all data sets were divided with a ratio of 7:3). The entire research process was conducted five times using five different training sets and test sets. The results showed that the model parameters obtained from different data sets had little change, indicating that the research was reproducible (Additional file 1: Table S1).

### Statistical analysis

All statistical analyses were used the two-side test. Continuous variables were tested by the t-test, and expressed as mean ± SD, or by the Mann–Whitney U-test, and presented as median (interquartile range). Categorical variables were analyzed by the Chi-square test (χ^2^ test), and displayed as a number (n) and percentage (%). *P* < 0.05 was considered as statistical significance. All analyses were performed using SAS (version 9.4), Python (version 3.7), and Scikit-learn (version 0.21).

Missing data in variables (such as drinking history, smoking history) were adopted by the random forest filling method. Continuous variables (age, CHA2DS2VASc score, HAS-BLED score, etc.) were processed for data standardization to eliminate dimensional effects. The data set was divided into the training set and test set based on the ratio of 7:3.

## Results

### Baseline characteristics

**Fig. 1:**
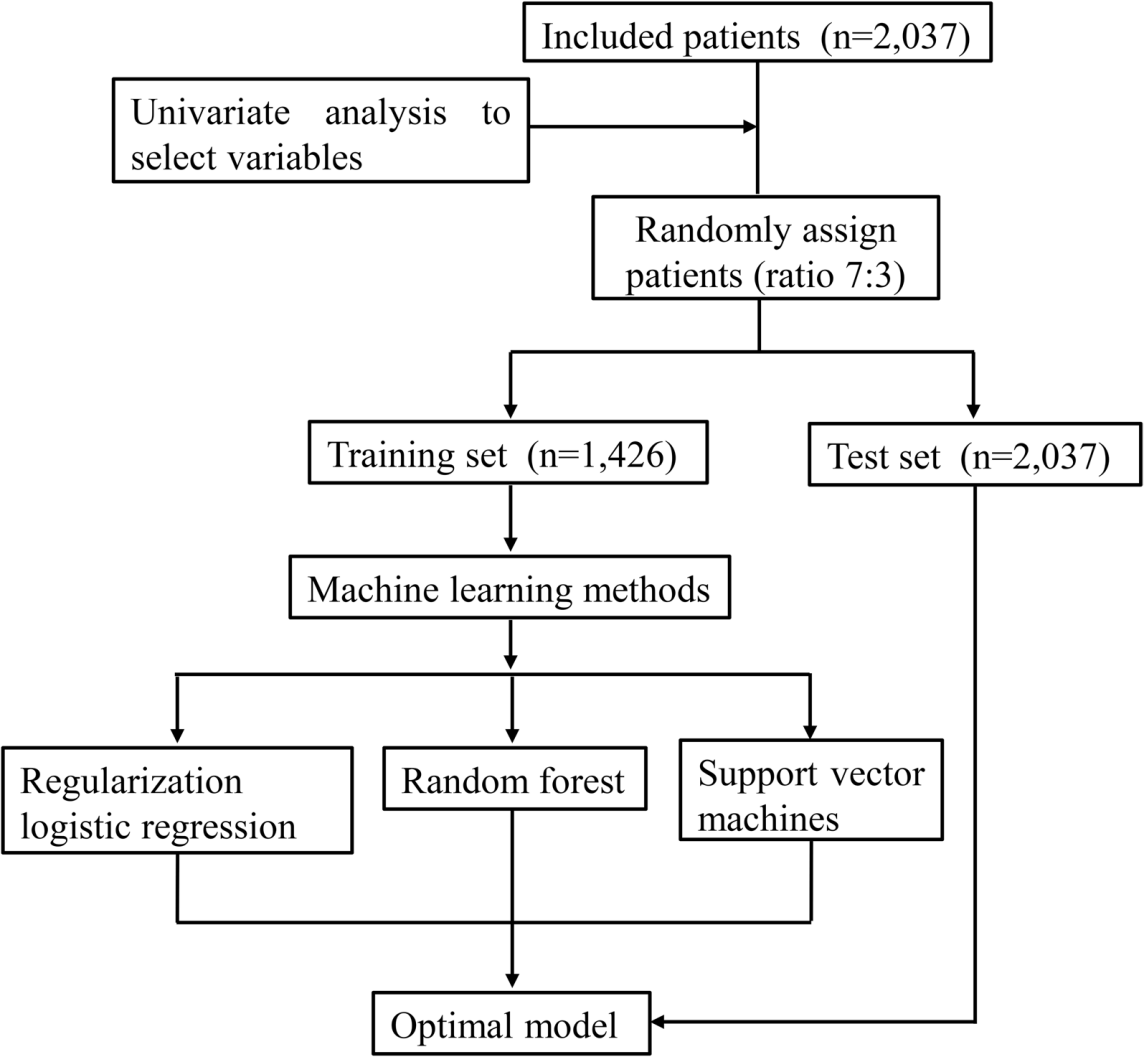
The flowchart of the study process

A total of 2037 CAD patients with AF were included in this study, with a mean age of 72.26 ± 10.40 years, the median number of hospitalizations was 1.00 (1.00, 2.00), the media CHA2DS2VASc score was 3 (2.00, 5.00), and the mean HAS-BLED score was 2.04 ± 1.14. Of these patients, 1128 (55.38%) were men, only 125 (6.14%) patients were treated with PCI. Among the types of AF, 27 (1.33%) were initial patients, 1115 (54.74%) were paroxysmal patients, 490 (24.05%) were persistent patients, and 405 (19.88%) were permanent patients. In the type of CAD, 555 (27.25%) patients were stable type, 1420 (69.71%) were unstable type, and 62 (3.04%) were acute myocardial infarction. The all-cause mortality of CAD patients with AF was 6.77% (138 cases). Baseline characteristics were shown in Table 1. The study flowchart was displayed in Fig. 1.

**Table 1:** Baseline characteristics and univariate analysis

Variables	Total (n = 2037)	All-cause mortality		Statistics	P*
		The survival group (n = 1899)	The death group (n = 138)		
Gender, n (%)				χ^2^ = 0.005	0.941
Male	1128 (55.38)	1052 (55.40)	76 (55.07)		
Female	909 (44.62)	847 (44.60)	62 (44.93)		
Age (years), mean ± SD	72.26 ± 10.40	71.71 ± 10.26	79.82 ± 9.28	t = − 9.804	< 0.001
The number of hospitalizations, M (Q_1_, Q_3_)	1.00 (1.00, 2.00)	1.00 (1.00, 2.00)	1.00 (1.00, 2.00)	Z = 1.780	0.075
Types of AF, n (%)				χ^2^ = 1.054	0.788
Initial	27 (1.33)	25 (1.32)	2 (1.45)		
Paroxysmal	1115 (54.74)	1045 (55.03)	70 (50.72)		
Persistent	490 (24.05)	455 (23.96)	35 (25.36)		
Permanent	405 (19.88)	374 (19.69)	31 (22.46)		
Types of CAD, n (%)				χ^2^ = 0.442	0.802
Stable	555 (27.25)	515 (27.12)	40 (28.99)		
Unstable	1420 (69.71)	1327 (69.88)	93 (67.39)		
Acute myocardial Infarction, n (%)	62 (3.04)	57 (3.00)	5 (3.62)		
Diabetes, n (%)				χ^2^ = 1.971	0.160
No	1490 (73.15)	1382 (72.78)	108 (78.26)		
Yes	547 (26.85)	517 (27.22)	30 (21.74)		
Hypertension, n (%)				χ^2^ = 0.011	0.915
No	791 (38.83)	738 (38.86)	53 (38.41)		
Yes	1246 (61.17)	1161 (61.14)	85 (61.59)		
Heart failure, n (%)				χ^2^ = 0.207	0.649
No	1306 (64.11)	1220 (64.24)	86 (62.32)		
Yes	731 (35.89)	679 (35.76)	52 (37.68)		
Cardiac function, n (%)				χ^2^ = 7.784	0.051
I	1081 (53.07)	1016 (53.50)	65 (47.10)		
II	497 (24.40)	468 (24.64)	29 (21.01)		
III	328 (16.10)	298 (15.69)	30 (21.74)		
IV	131 (6.43)	117 (6.16)	14 (10.14)		
Peripheral vascular diseases, n (%)				χ^2^ = 1.957	0.162
No	1834 (90.03)	1705 (89.78)	129 (93.48)		
Yes	203 (9.97)	194 (10.22)	9 (6.52)		
Ischemia stroke, n (%)				χ^2^ = 7.101	0.008
No	1496 (73.44)	1408 (74.14)	88 (63.77)		
Yes	541 (26.56)	491 (25.86)	50 (36.23)		
Bleeding history, n (%)				χ^2^ = 10.768	0.001
No	1980 (97.20)	1852 (97.53)	128 (92.75)		
Yes	57 (2.80)	47 (2.47)	10 (7.25)		
Peptic ulcer, n (%)				χ^2^ = 0.439	0.508
No	1991 (97.74)	1855 (97.68)	136 (98.55)		
Yes	46 (2.26)	44 (2.32)	2 (1.45)		
Drinking history, n (%)				χ^2^ = 10.744	0.001
No	297 (14.58)	290 (15.27)	7 (5.07)		
Yes	1740 (85.42)	1609 (84.73)	131 (94.93)		
Smoking history, n (%)				χ^2^ = 5.966	0.015
No	433 (21.26)	415 (21.85)	18 (13.04)		
Yes	1604 (78.74)	1484 (78.15)	120 (86.96)		
Cardioversion, n (%)				χ^2^ = 10.676	0.001
No	1589 (78.01)	1466 (77.20)	123 (89.13)		
Yes	448 (21.99)	433 (22.80)	15 (10.87)		
PCI, n (%)				χ^2^ = 5.646	0.018
No	1912 (93.86)	1776 (93.52)	136 (98.55)		
Yes	125 (6.14)	123 (6.48)	2 (1.45)		
CHA_2_DS_2_VASc, M (Q_1_, Q_3_)	3.00 (2.00, 5.00)	3.00 (2.00, 4.00)	4.00 (3.00, 5.00)	Z = 3.457	< 0.001
HAS-BLED, M (Q_1_, Q_3_)	2.04 ± 1.14	2.02 ± 1.14	2.39 ± 1.14	t = 3.726	< 0.001
*In-hospital medication*					
Aspirin, n (%)				χ^2^ = 9.499	0.002
No	727 (35.69)	661 (34.81)	66 (47.83)		
Yes	1310 (64.31)	1238 (65.19)	72 (52.17)		
Clopidogrel, n (%)				χ^2^ = 2.924	0.087
No	1294 (63.52)	1197 (63.03)	97 (70.29)		
Yes	743 (36.48)	702 (36.97)	41 (29.71)		
Ticagrelor, n (%)				χ^2^ = 0.541	0.462
No	2019 (99.12)	1883 (99.16)	136 (98.55)		
Yes	18 (0.88)	16 (0.84)	2 (1.45)		
Warfarin, n (%)				χ^2^ = 6.279	0.012
No	1432 (70.30)	1322 (69.62)	110 (79.71)		
Yes	605 (29.70)	577 (30.38)	28 (20.29)		
Dabigatran, n (%)				χ^2^ = 2.138	0.144
No	2008 (98.58)	1870 (98.47)	138 (100.00)		
Yes	29 (1.42)	29 (1.53)	0 (0.00)		
Rivaroxaban, n (%)				χ^2^ = 0.022	0.883
No	2020 (99.17)	1883 (99.16)	137 (99.28)		
Yes	17 (0.83)	16 (0.84)	1 (0.72)		
ACEI/ARB, n (%)				χ^2^ = 2.820	0.093
No	1070 (52.53)	988 (52.03)	82 (59.42)		
Yes	967 (47.47)	911 (47.97)	56 (40.58)		
Beta-blockers, n (%)				χ^2^ = 12.093	< 0.001
No	766 (37.60)	695 (36.60)	71 (51.45)		
Yes	1271 (62.40)	1204 (63.40)	67 (48.55)		
Lipid-lowing treatment, n (%)				χ^2^ = 17.522	< 0.001
No	424 (20.81)	376 (19.80)	48 (34.78)		
Yes	1613 (79.19)	1523 (80.20)	90 (65.22)		
Diuretic, n (%)				χ^2^ = 0.673	0.412
No	969 (47.57)	908 (47.81)	61 (44.20)		
Yes	1068 (52.43)	991 (52.19)	77 (55.80)		
Digoxin, n (%)				χ^2^ = 0.866	0.352
No	1372 (67.35)	1284 (67.61)	88 (63.77)		
Yes	665 (32.65)	615 (32.39)	50 (36.23)		
Nitrates, n (%)				χ^2^ = 1.940	0.164
No	1020 (50.07)	943 (49.66)	77 (55.80)		
Yes	1017 (49.93)	956 (50.34)	61 (44.20)		
Trimetazidine, n (%)				χ^2^ = 0.785	0.376
No	1362 (66.86)	1265 (66.61)	97 (70.29)		
Yes	675 (33.14)	634 (33.39)	41 (29.71)		
Amiodarone, n (%)				χ^2^ = 2.811	0.094
No	1646 (80.81)	1527 (80.41)	119 (86.23)		
Yes	391 (19.19)	372 (19.59)	19 (13.77)		
Propafenone, n (%)				χ^2^ = 0.106	0.745
No	2000 (98.18)	1865 (98.21)	135 (97.83)		
Yes	37 (1.82)	34 (1.79)	3 (2.17)		
CCB, n (%)				χ^2^ = 1.867	0.172
No	1415 (69.46)	1312 (69.09)	103 (74.64)		
Yes	622 (30.54)	587 (30.91)	35 (25.36)		
Thrombolysis, n (%)				-	1.000
No	2029 (99.61)	1891 (99.58)	138 (100.00)		
Yes	8 (0.39)	8 (0.42)	0 (0.00)		
Fondaparinux sodium, n (%)				χ^2^ = 5.021	0.025
No	1990 (97.69)	1859 (97.89)	131 (94.93)		
Yes	47 (2.31)	40 (2.11)	7 (5.07)		
Low-molecular-weight heparin, n (%)				χ^2^ = 10.591	0.001
No	1502 (73.74)	1384 (72.88)	118 (85.51)		
Yes	535 (26.26)	515 (27.12)	20 (14.49)		
Tirofiban, n (%)				χ^2^ = 0.461	0.497
No	2009 (98.63)	1872 (98.58)	137 (99.28)		
Yes	28 (1.37)	27 (1.42)	1 (0.72)		
PPI, n (%)				χ^2^ = 0.473	0.491
No	1393 (68.38)	1295 (68.19)	98 (71.01)		
Yes	644 (31.62)	604 (31.81)	40 (28.99)		
In-hospital bleeding, n (%)				-	0.005
No	2026 (99.46)	1892 (99.63)	134 (97.10)		
Yes	11 (0.54)	7 (0.37)	4 (2.90)		
Embolism in-hospital, n (%)				χ^2^ = 0.837	0.360
No	2021 (99.21)	1885 (99.26)	136 (98.55)		
Yes	16 (0.79)	14 (0.74)	2 (1.45)		
*Out-of-hospital medication*					
Aspirin, n (%)				χ^2^ = 8.295	0.004
No	926 (45.46)	847 (44.60)	79 (57.25)		
Yes	1111 (54.54)	1052 (55.40)	59 (42.75)		
Clopidogrel, n (%)				χ^2^ = 6.174	0.013
No	1516 (74.42)	1401 (73.78)	115 (83.33)		
Yes	521 (25.58)	498 (26.22)	23 (16.67)		
Ticagrelor, n (%)				χ^2^ = 0.022	0.883
No	2020 (99.17)	1883 (99.16)	137 (99.28)		
Yes	17 (0.83)	16 (0.84)	1 (0.72)		
Warfarin, n (%)				χ^2^ = 5.724	0.017
No	1507 (73.98)	1393 (73.35)	114 (82.61)		
Yes	530 (26.02)	506 (26.65)	24 (17.39)		
Dabigatran, n (%)				χ^2^ = 1.102	0.294
No	1978 (97.10)	1842 (97.00)	136 (98.55)		
Yes	59 (2.90)	57 (3.00)	2 (1.45)		
Rivaroxaban, n (%)				χ^2^ = 0.461	0.497
No	2009 (98.63)	1872 (98.58)	137 (99.28)		
Yes	28 (1.37)	27 (1.42)	1 (0.72)		
ACEI/ARB, n (%)				χ^2^ = 4.185	0.041
No	1174 (57.63)	1083 (57.03)	91 (65.94)		
Yes	863 (42.37)	816 (42.97)	47 (34.06)		
Beta-blockers, n (%)				χ^2^ = 20.436	< 0.001
No	908 (44.58)	821 (43.23)	87 (63.04)		
Yes	1129 (55.42)	1078 (56.77)	51 (36.96)		
Statins, n (%)				χ^2^ = 27.907	< 0.001
No	504 (24.74)	444 (23.38)	60 (43.48)		
Yes	1533 (75.26)	1455 (76.62)	78 (56.52)		
Diuretic, n (%)				χ^2^ = 1.808	0.179
No	1203 (59.06)	1129 (59.45)	74 (53.62)		
Yes	834 (40.94)	770 (40.55)	64 (46.38)		
Digoxin, n (%)				χ^2^ = 0.467	0.494
No	1540 (75.60)	1439 (75.78)	101 (73.19)		
Yes	497 (24.40)	460 (24.22)	37 (26.81)		
Nitrates, n (%)				χ^2^ = 4.213	0.040
No	1296 (63.62)	1197 (63.03)	99 (71.74)		
Yes	741 (36.38)	702 (36.97)	39 (28.26)		
Trimetazidine, n (%)				χ^2^ = 2.726	0.099
No	1518 (74.52)	1407 (74.09)	111 (80.43)		
Yes	519 (25.48)	492 (25.91)	27 (19.57)		
Amiodarone, n (%)				χ^2^ = 4.672	0.031
No	1785 (87.63)	1656 (87.20)	129 (93.48)		
Yes	252 (12.37)	243 (12.80)	9 (6.52)		
Propafenone, n (%)				χ^2^ = 0.745	0.388
No	2004 (98.38)	1867 (98.31)	137 (99.28)		
Yes	33 (1.62)	32 (1.69)	1 (0.72)		

*P-value showed the comparison result between the survival group and the death group; *CCB* calcium channel blockers, *ACEI/ARB* angiotensin converting enzyme inhibitor/Angiotensin II receptor blockers, *PPI* proton pump inhibitors

### Comparison of the survival group and the death group

The univariate analysis showed that age (t =  − 9.804, *P* < 0.001), CHA2DS2VASc score (Z = 3.457, *P* = 0.005), HAS-BLED score (t = 3.726, *P* < 0.001), and the proportion of ischemic stroke (χ^2^ = 7.101, *P* = 0.008), bleeding history (χ^2^ = 10.768, *P* = 0.001), drinking history (χ^2^ = 10.744, *P* = 0.001), smoking history (χ^2^ = 5.966, *P* = 0.015), in-hospital bleeding (t = 3.726, *P* < 0.001), in-hospital medication of fondaparinux sodium (χ^2^ = 5.021, *P* = 0.025) of the death group were significantly higher than those of the survival group.

In addition, compared with the death group, the proportion of cardioversion (χ^2^ = 10.676, *P* = 0.001), PCI treatment (χ^2^ = 5.646, *P* = 0.018), in-hospital medication (such as aspirin (χ^2^ = 9.499, *P* = 0.002), warfarin (χ^2^ = 6.279, *P* = 0.012), beta-blockers (χ^2^ = 12.093, *P* < 0.001), lipid-lowering drugs (χ^2^ = 17.522, *P* < 0.001), and low-molecular-weight heparin (χ^2^ = 10.591, *P* = 0.001)), and out-of-hospital medication (such as aspirin (χ^2^ = 8.295, *P* = 0.004), clopidogrel (χ^2^ = 6.174, *P* = 0.013), warfarin (χ^2^ = 5.724, *P* = 0.017), ACEI/ARB (χ^2^ = 4.185, *P* = 0.041), beta-blockers (χ^2^ = 20.436, *P* < 0.001), statins (χ^2^ = 27.907, *P* < 0.001), nitrates (χ^2^ = 4.213, *P* = 0.040), and amiodarone (χ^2^ = 4.672, *P* = 0.031)) were higher in the survival group (Table 1).

### Variable importance

**Fig. 2:**
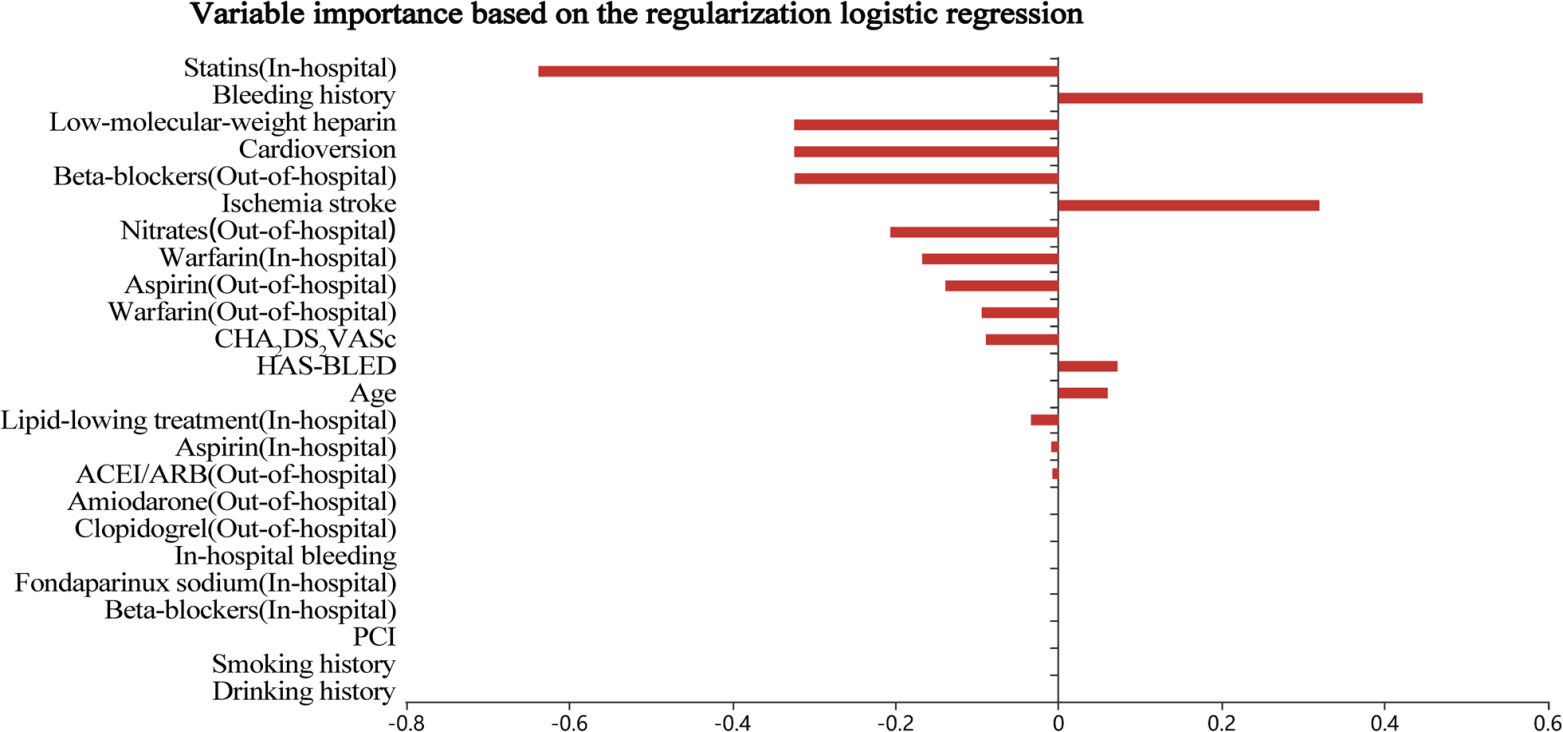
The importance of variables based on the regularization logistic regression model

**Fig. 3:**
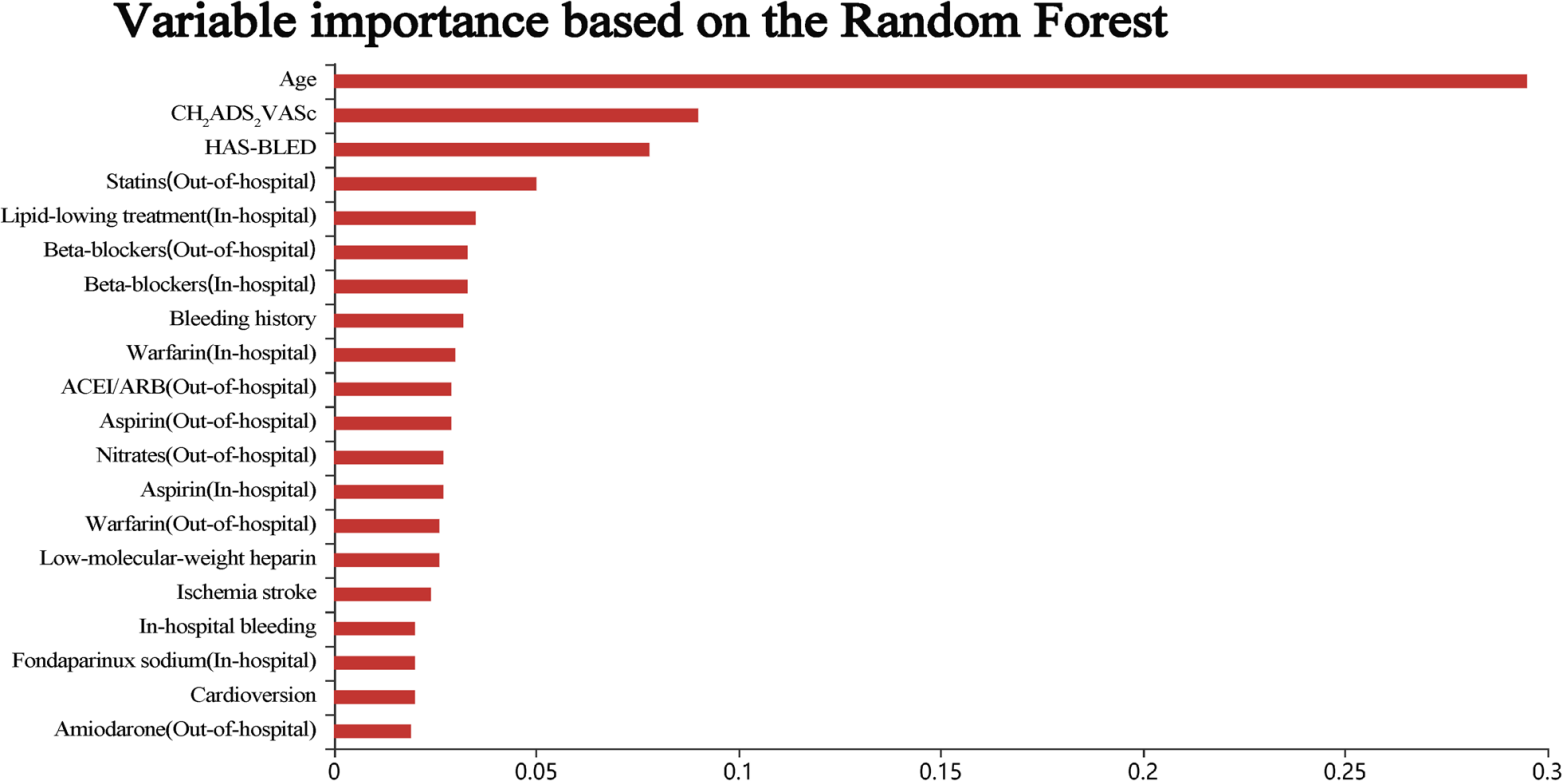
The importance of variables in the random forest model

**Fig. 4:**
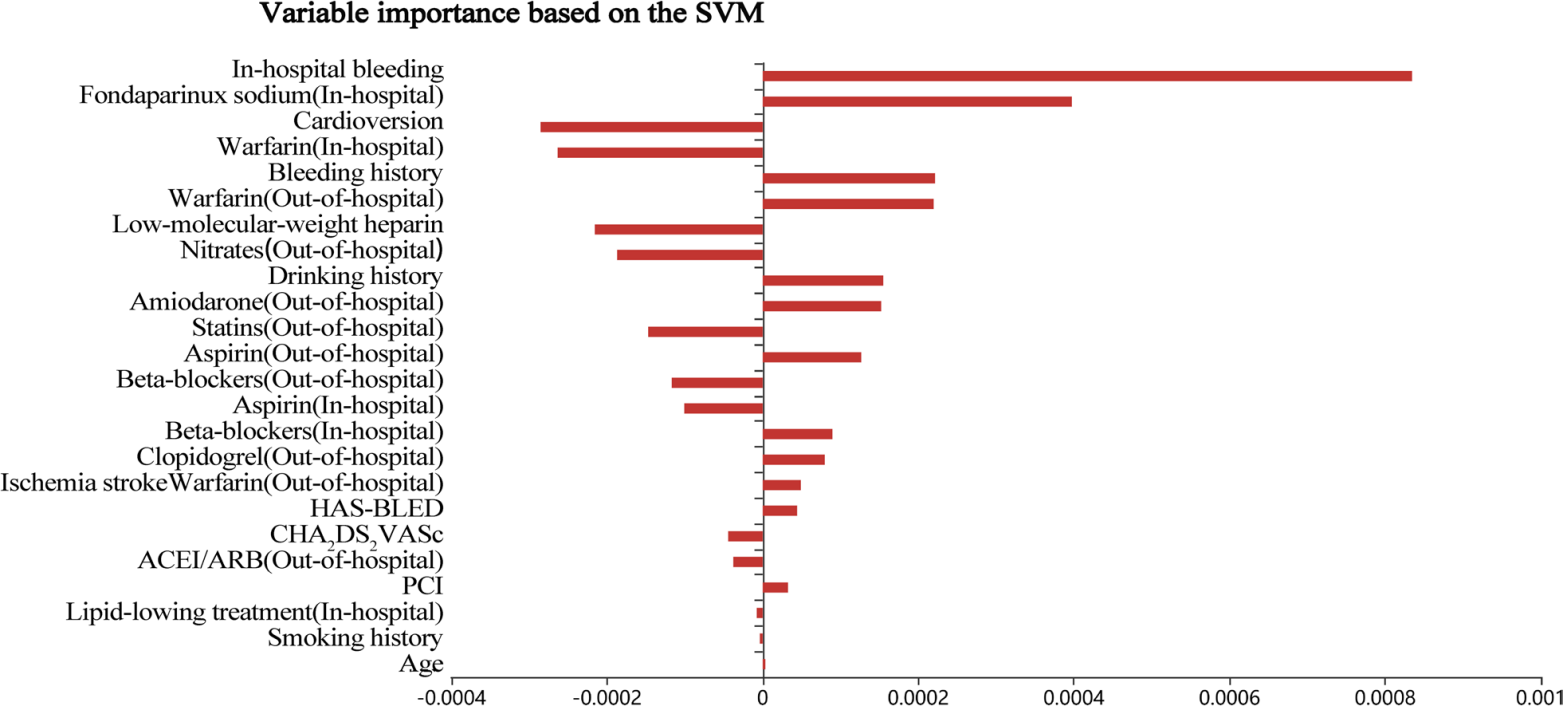
The importance of variables based on the support vector machines model

After univariate analysis, 24 variables with statistical differences were included in the predictive model. The model coefficient was used to evaluate the importance of variables in the regularization logistic regression model and support vector machines model, while the Gini importance index was used for evaluation in the random forest model. The importance of variables based on the regularization logistic regression model was shown in Fig. 2. The main predictors for the regularization logistic regression model were as follows: out-of-hospital medication (statins, beta-blockers, nitrates, aspirin, and warfarin), bleeding history, in-hospital medication (low-molecular-weight heparin and warfarin), cardioversion, ischemic stroke, CHA2DS2VASc score, HAS-BLED score, and age, etc. The important variables for the random forest model were age, CHA2DS2VASc score, HAS-BLED score, out-of-hospital medication (statins and beta-blockers), in-hospital medication (lipid-lowering drugs, beta-blockers, and warfarin), and bleeding history, etc. (Fig. 3). Patient’s in-hospital bleeding, in-hospital medication (fondaparinux sodium, warfarin, and low-molecular-weight heparin), cardioversion, bleeding history, out-of-hospital medication (warfarin, nitrates, amiodarone, and statins), and drinking history, etc. were the important variables for the support vector machines model (Fig. 4).

### Model performance comparison

The performance of the three models in the training set was summarized (Table 2). The regularization logistic regression model had the highest AUC (0.788; 95% CI 0.743–0.833) and specificity (0.708; 95% CI 0.683–0.733). The AUC of the random forest model and support vector machines model was 0.744 (95% CI 0.693–0.795) and 0.689 (95% CI 0.635–0.744), respectively. The performance of the three models in the test set shown in Table 3, the AUC of regularization logistic regression model, random forest model, and support vector machines model was 0.732 (95% CI 0.649–0.816), 0.728 (95% CI 0.642–0.813), and 0.712 (95% CI 0.630–0.794), respectively. The results of the models on the test set showed that the three models fit well.

**Table 2:** The performance of the three models in the trainig set

Models	Sensitivity (95% CI)	Specificity (95% CI)	Accuracy	AUC (95% CI)
Regularization logistic regression	0.786 (0.691–0.862)	0.708 (0.683–0.733)	0.932	0.788 (0.743–0.833)
Random forest	0.806 (0.714–0.879)	0.601 (0.574–0.628)	0.931	0.744 (0.693–0.795)
Support vector machines	0.612 (0.508–0.709)	0.680 (0.654–0.705)	0.931	0.689 (0.635–0.744)

*AUC* area under the curve, *CI* confidence intervals

**Table 3:** The performance of the three models in the test set

Models	Sensitivity (95% CI)	Specificity (95% CI)	Accuracy	AUC (95% CI)
Regularization logistic regression	0.725 (0.561–0.854)	0.699 (0.660–0.737)	0.936	0.732 (0.649–0.816)
Random forest	0.750 (0.588–0.873)	0.663 (0.622–0.701)	0.935	0.728 (0.642–0.813)
Support vector machines	0.675 (0.509–0.814)	0.668 (0.628–0.706)	0.935	0.712 (0.630–0.794)

**Fig. 5:**
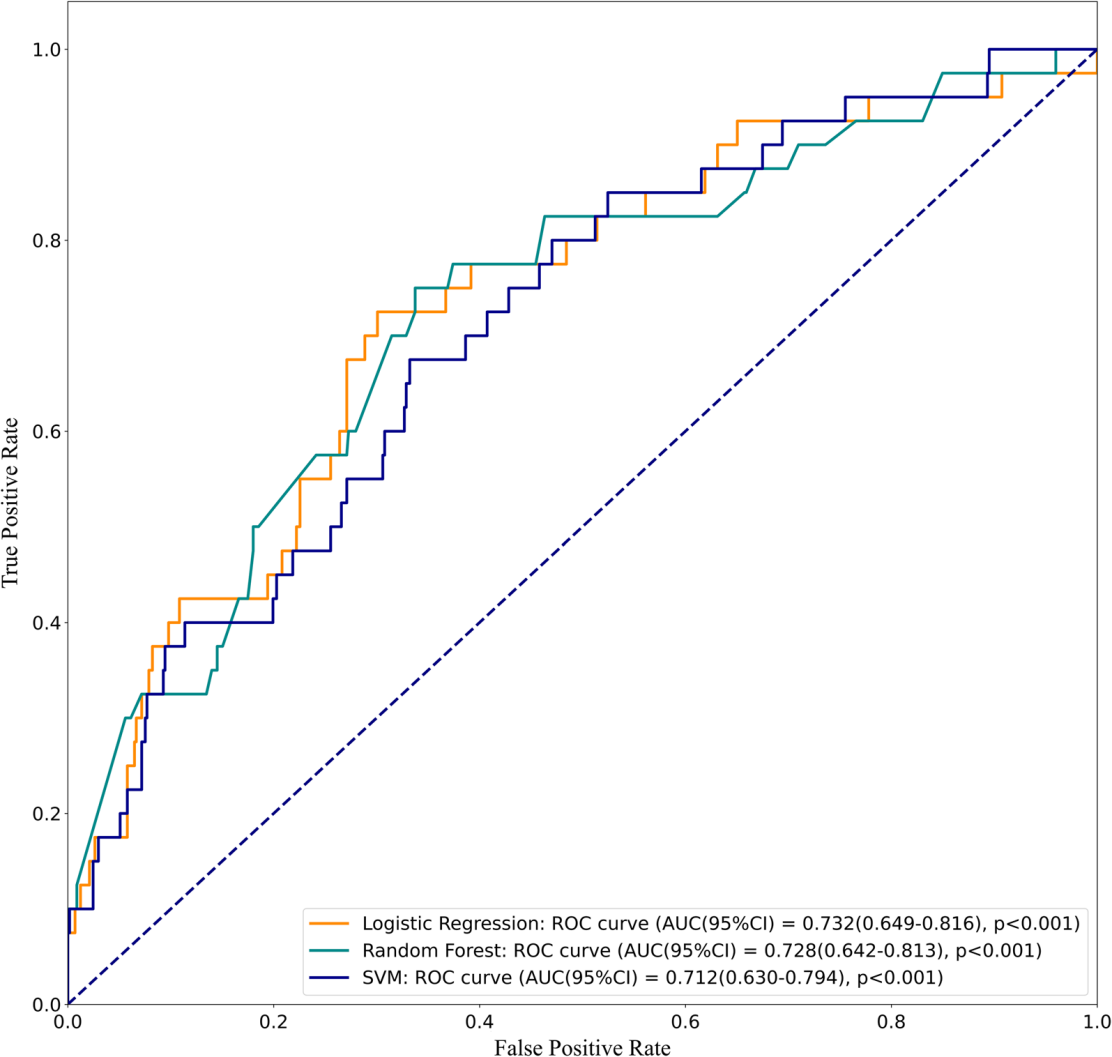
The difference of the receiver operating characteristic (ROC) curves among the three models

According to the result of the DeLong test [15], the receiver operating characteristic (ROC) curve of the three models was analyzed for differences. As demonstrated in Fig. 5, no statistical difference was observed in the ROC curve (regularization logistic regression model vs. random forest model, *P* = 0.888; regularization logistic regression model vs. support vector machines model, *P* = 0.554; random forest model vs. support vector machines model, *P* = 0.724).

## Discussion

In this study, three ML methods were used to predict the all-cause mortality in CAD patients with AF. The AUC of the regularization logistic regression model, random forest model, and support vector machines model was 0.732, 0.728, and 0.712, respectively. The regularization logistic regression model had the highest AUC value, specificity, and accuracy among the three models. However, the ROC curve of the three models had no significant difference. Although the three models had similar predictive capabilities, the regularization logistic regression model was recommended to be used in clinical practice, because it was simpler and more interpretable.

ML method is a form of artificial intelligence, and does not make a priori assumptions about causality, which distinguishes it from regression-based methods. ML had been widely used in the diagnosis and prognosis of CAD [12, 16, 17]. However, no studies developed a ML prediction model that can be used to predict all-cause mortality in CAD patients with AF. Our study provided three ML models to predict all-cause mortality in patients with CAD and AF. The AUC of the regularization logistic regression model was 0.732, which was the best among the three models. The study of Reeh et al. provided a more accurate model to predict the possibility of CAD based on the Diamon–Forrester prediction model [18]. Motwani et al. performed a ML model to predict 5-year all-cause mortality in patients with CAD. Their studies showed that ML combining clinical and coronary computed tomographic angiography data to predict 5-year all-cause mortality was found to be significantly better than existing clinical or coronary computed tomographic angiography metrics alone [9]. Existing studies have suggested that some biomarkers may predict the death of patients with CAD. Wada et al. presented that levels of vascular endothelial growth factor-C (VEGF-C) were inversely associated with all-cause mortality of CAD patients, and a low VEGF-C value may independently predict all-cause mortality [19]. In the study of Song et al., the increased risk of incident all-cause mortality was associated with higher baseline circulating 7-Ketocholesterol levels among CAD patients with stable conditions [20]. In the second prevention settings of CAD, Karakas et al. indicated that the single miRNAs derived from peripheral blood can be used as a biomarker to predict the mortality of CAD patients [21]. In future studies, researchers may try to incorporate some important biomarkers into the prediction model to obtain a better model.

In our three predictive models, the important variables of each model were different. For the regularization logistic regression model, out-of-hospital medication (statins, beta-blockers), bleeding history, etc. were more important. However, the important variables in the random forest model were patient’s age, CHA2DS2VASc score, HAS-BLED score, etc. The important variables in the support vector machines model were in-hospital bleeding, in-hospital medication of fondaparinux sodium and warfarin, etc. The difference in important variables among the three models was caused by the limitation of ML. More and more variables and interactions were used in ML to predict risk, but specific treatment goals that can reduce the risk may be difficult to determine [9]. However, some important variables in the three models were consistent, such as medication (statins, beta-blockers, warfarin, and low-molecular-weight heparin), bleeding history, and cardioversion. These variables should be noticed in clinical practice. A systematic meta-analysis showed that statin therapy was beneficial for the prevention of AF in CAD patients [22]. Joseph et al. conducted a systematic review that beta-blockers had important values in reducing the mortality and morbidity of myocardial infarction in CAD patients [23].

To the best of our knowledge, this was the first ML model to predict all-cause mortality of CAD patients with AF. In addition, we provided three prediction models based on three ML methods. The regularization logistic regression model had good predictive ability and was recommended to be used. The regularization logistic regression model may provide a tool to predict the all-cause mortality of CAD patients with AF, and provide clinicians with early intervention for patients who may be at high risk of mortality, which has important clinical significance for improving the prognosis of patients. However, this study had some limitations. First, the randomness of the selection of different model variables in ML cannot derive consistent important variables, which will bring difficulties in the prevention and treatment of disease. Second, the model of internal validation fit well, but external validation of the prediction models was necessary. Third, the sample size of this study was not larger, and future studies may require larger sample sizes to provide more reliable results.

## Conclusion

This study used three models to predict the all-cause mortality of CAD patients with AF based on ML methods. No significant difference was observed in the three models. Combining the performance of all aspects of the models, the regularization logistic regression model was recommended to be used in clinical practice. A better model based on large samples and multiple centers will be needed in future studies.

## Supplementary Information

**Additional file 1.** Sample size and reproducibility analysis.

## Data Availability

The datasets generated and analyzed during the current study are available from the corresponding author on reasonable request.

## Abbreviations

ML: Machine learning
CAD: Coronary artery disease
AF: Atrial fibrillation
AUC: Area under the curve
CI: Confidence intervals
WHO: World Health Organization
PCI: Percutaneous coronary intervention
