# Supplementary material for: Prediction of all-cause mortality in coronary artery disease patients with atrial fibrillation based on machine learning models
Source: BMC Cardiovasc Disord. 2021 Oct 16;21:499. doi: 10.1186/s12872-021-02314-w (PMC8520292; doi:10.1186/s12872-021-02314-w)
Supplement: Supplementary file 1 — Additional file 1. Sample size and reproducibility analysis. [file 12872_2021_2314_MOESM1_ESM.docx]

a
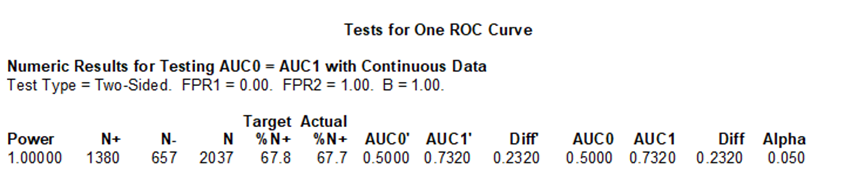


b


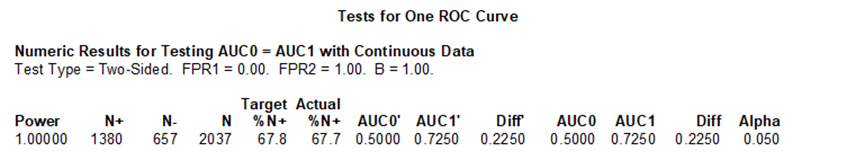


c


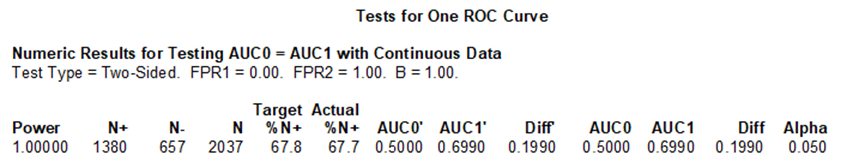


d


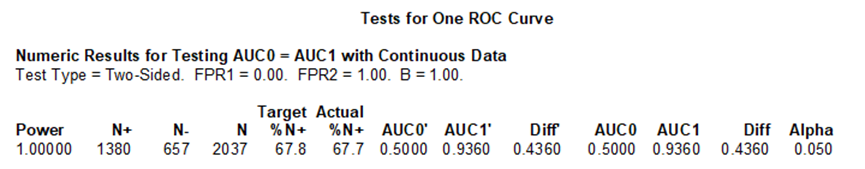


**Supplement Figure 1**. The statistical power of the regularization logistic regression model performance indicators. (a) The statistical power of the area under the curve (AUC); (b) The statistical power of the sensitivity; (c) The statistical power of the specificity; (d) The statistical power of the accuracy.

**Supplement Table 1**. The performance of the regularization logistic regression model in the test set of different random number seeds.

| **Random number seeds** | **Sensitivity**  **(95%CI)** | **Specificity**  **(95%CI)** | **Accuracy** | **AUC (95%CI)** |
| --- | --- | --- | --- | --- |
| 9 | 0.684 (0.676-0.692) | 0.814 (0.806-0.822) | 0.941 | 0.763 (0.675-0.851) |
| 8 | 0.667 (0.659-0.675) | 0.734 (0.726-0.742) | 0.925 | 0.734 (0.668-0.799) |
| 7 | 0.769 (0.761-0.777) | 0.628 (0.620-0.636) | 0.935 | 0.757 (0.683-0.832) |
| 5 | 0.628 (0.620-0.636) | 0.789 (0.781-0.797) | 0.930 | 0.737 (0.657-0.817) |
| 3 | 0.646 (0.638-0.654) | 0.791 (0.782-0.799) | 0.925 | 0.736 (0.655-0.817) |
